# Piceatannol as a safe senotherapeutic: ameliorating senescence-associated markers in a radiation-induced murine model

**DOI:** 10.1038/s41420-026-03253-1

**Published:** 2026-07-14

**Authors:** Alessia Ambrosino, Claudia Moriello, Nicola Alessio, Domenico Aprile, Salvatore Cappabianca, Nicolamaria Tarantino, Gianfranco Peluso, Giovanni Di Bernardo, Umberto Galderisi

**Affiliations:** 1 Department of Experimental Medicine, University of Campania “Luigi Vanvitelli”, Naples, Italy; 2 Department of Life Sciences, Health and Health Professions, Link Campus University, Rome, Italy; 3 Department of Precision Medicine, University of Campania “Luigi Vanvitelli”, Naples, Italy; 4 Saint Camillus International University of Health and Medical Sciences, Rome, Italy; 5 Genome and Stem Cell Center (GENKÖK) Erciyes University, Kayseri, Turkey; 6 Sbarro Institute for Cancer Research and Molecular Medicine, Center for Biotechnology, Temple University, Philadelphia, PA USA

**Keywords:** Senescence, Experimental models of disease

## Abstract

Cellular senescence is a key driver of age-associated tissue dysfunction through the secretion of pro-inflammatory and pro-senescent factors (SASP). Among these, insulin-like growth factor binding proteins (IGFBPs) have emerged as causal mediators of senescence propagation. Piceatannol (PCT), a naturally occurring stilbene and resveratrol metabolite with superior pharmacokinetic properties, exhibits senomorphic activity in vitro, but its in vivo senotherapeutic potential remains poorly defined. We established a murine model of mild aging using sublethal X-ray irradiation, in which organ function remains largely preserved but molecular and cellular hallmarks of aging, such as senescent cell accumulation, SASP factor elevation, tissue remodeling, and low-grade inflammation, are detectable. This controlled model mimics the early stage of aging seen in many healthy middle-aged individuals, particularly prevalent in Western societies, and provides a relevant platform for testing preventive senotherapeutics. Mice were treated with oral PCT at a human-translatable dose and evaluated for behavioral performance, circulating SASP factors, tissue senescence, inflammation and fibrosis. PCT significantly improved motor coordination and spatial memory while reducing systemic inflammation and circulating SASP factors, including the senescence-propagating IGFBPs IGFBP4, IGFBP5, and IGFBP7. In kidney and heart, the organs most affected by radiation-induced aging, PCT reduced senescent cell burden, CD68⁺ inflammatory foci, fibrotic remodeling, and the senescence markers P53 and P21. PCT also preserved mesenchymal stromal cell clonogenic capacity. These multi-organ benefits were achieved without detectable toxicity in healthy animals. Our findings position PCT as a promising, safe, nutritionally derived senotherapeutic candidate for delaying aging-associated deterioration. This mild aging model, capturing preclinical senescence without overt organ failure, may accelerate the development of interventions aimed at preserving health span in middle-aged populations.

## Introduction

Over the past century, human life expectancy has increased significantly, yet this gain in lifespan has not been matched by equivalent improvements in health span, the period of life spent in good health during old age. Aging is a complex, multifactorial process marked by molecular and cellular deterioration that progressively impairs organ function, leading to increased frailty, susceptibility to diseases, and mortality [1, 2].

Healthy aging involves continuously optimizing opportunities to preserve and enhance physical and mental well-being, autonomy, and overall quality of life throughout an individual’s lifespan. It depends on dynamic and successful interactions between biological, psychological, and environmental factors, with aging being influenced by a combination of genetic predisposition, environmental exposures, and stochastic events [1–3].

Given this understanding, researchers are developing strategies to prevent or delay aging [3, 4]. In this context, senescent cells have emerged as a key target for potentially delaying or mitigating the adverse effects of aging. Over time, damage accumulates in cellular macromolecules, inducing senescence in otherwise healthy cells, which subsequently lose their normal physiological functions [5–7].

Senescent cells adopt new, dysfunctional activities, largely driven by the secretion of the senescence-associated secretory phenotype (SASP). SASP comprises numerous factors acting through autocrine, paracrine, or endocrine pathways to reinforce senescence, induce secondary senescence in healthy cells, promote inflammation, and, depending on the microenvironment, either inhibit or promote tumorigenesis. Multiple studies have demonstrated a direct link between cellular senescence and aging [7].

Among SASP components, insulin-like growth factor binding proteins (IGFBPs) have emerged as key drivers of senescence propagation. We identified IGFBP4 as the first circulating SASP factor capable of inducing senescence in distant organs via ROS–prostaglandin–EP2 signaling, establishing senescence as a systemic, endocrine-like process. We later showed that IGFBP5 triggers senescence through nuclear activation of retinoic acid–dependent transcriptional programs, while IGFBP7 promotes a pro-senescent metabolic environment by disrupting insulin, IGF-1, and activin signaling [8–10]. Together, these three IGFBPs form a coordinated pro-senescent network with direct translational relevance as circulating biomarkers of senescence propagation.

Landmark research has shown that selectively clearing senescent cells in aged mice extends health span and reduces deterioration across multiple organs and tissues. Accordingly, a new class of drugs termed senotherapeutics is attracting attention for its potential to promote healthy aging. Senotherapeutics include two main types: senolytics and senomorphics. Senolytics specifically target and eliminate senescent cells by exploiting their heightened resistance to apoptosis. Senomorphics, in contrast, can either prevent the onset of senescence in response to genotoxic stress by disrupting senescence-triggering signals or suppress the harmful effects of the SASP. Some senomorphics may even partially reverse the senescent phenotype [11–15].

Polyphenols, plant-derived secondary metabolites, have been used for centuries for their diverse effects on living organisms, including medicinal benefits. Recently, they have been investigated as potential senotherapeutics in various experimental models. Many polyphenols display senomorphic properties by suppressing SASP factors released by different types of senescent cells, while some also exhibit senolytic activity by inducing apoptosis in senescent cells [16–18].

While resveratrol has been widely studied for its anti-aging potential, its clinical translation is limited by poor bioavailability (~1% in humans), rapid metabolism, and a short plasma half-life, which require high doses and may cause adverse effects [19, 20]. Piceatannol (PCT) (3,5,4′,3′-tetrahydroxystilbene), a natural metabolite of resveratrol found in grapes, berries, and passion fruit, contains an additional hydroxyl group at the 3′ position that confers superior pharmacokinetic properties. Compared with resveratrol, PCT shows higher cellular uptake, a longer plasma half-life (~8–12 h vs 2–4 h), greater antioxidant capacity, and increased stability under physiological conditions [21, 22]. Importantly, PCT is directly obtained from dietary sources without requiring metabolic conversion. Thus, PCT represents a next-generation polyphenol that may overcome the pharmacokinetic limitations of resveratrol while preserving its beneficial biological activities.

Our previous in vitro work demonstrated that PCT exhibits potent senomorphic activity in mesenchymal stromal cells (MSC), reducing SASP secretion and preserving stemness [23].

We therefore sought to evaluate the in vivo senotherapeutic potential of PCT in X-ray-irradiated mice, a controlled model of aging. We chose irradiated mice rather than naturally aged mice due to the high variability in aging-related changes, which complicates senotherapeutic studies. Chronological age does not always correlate with biological age, and rates of aging vary both between individuals and among organs within the same organism. By using irradiation to induce aging hallmarks at a defined starting point, we created a reproducible model for assessing progression and therapeutic intervention.

## Results

### Establishment of the irradiated mouse model for aging

Significant differences in radiosensitivity among inbred mouse strains have been well documented [24]. For this study, C57BL/6 J inbred mice were selected based on their demonstrated capacity for recovery following sublethal X-ray irradiation. Animals received a single 5 Gy dose of total body irradiation, with aging and senescence markers subsequently evaluated.

Aging progression varies not only between individuals but also among organs, cell types, subcellular organelles, and even macromolecules [1, 2, 25, 26]. Given this complexity, identifying an optimal set of criteria to assess aging, and thereby define healthy aging, is challenging [26].

A comprehensive evaluation requires consideration of both organismal-level characteristics (e.g., stress resistance, functional physiology of cardiovascular, renal, pulmonary, immune, and neurological systems) and cellular-level changes (e.g., senescence accumulation and related phenomena). In our model, we prioritized specific parameters for targeted investigation. Table 1 summarizes the parameters selected for the evaluation of our aging model.

**Table 1:** The Table shows the selected parameters for the evaluation of our aging model.

Category	Analysis	Parameter/Deficit
Behavioral tests	Rotarod test	Motor coordination
	Weight-lift test	Muscle strength
	Y-maze test	Spatial learning & memory
Biochemical analyses (ELIZA/Metabolic)	Cystatin C	Kidney function
	Creatinine	Kidney function
	Urea	Kidney function
	Albumin	Liver function
	AST	Liver function
	NT-proBNP	Cardiac stress
	Troponin C	Cardiac damage
	TNF-α	Inflammation (SASP)
	IL-6	Inflammation (SASP)
	CRP	Inflammation
	GDF15	Inflammation
	IGFBP4 - 5 - 7	SASP
Phenotypic & functional analyses	% fur gray area	Coat color phenotype
	CFU assay	Bone marrow clonogenic capacity
Histological analyses	SA-β-gal	Tissue senescence
	Masson’s trichrome	Fibrosis
	CD68	Macrophage infiltration
Molecular analyses (Western blot)	P53	Cellular senescence
	P21	Cellular senescence
	P16	Cellular senescence
	RB1	Cellular senescence
Molecular analyses (RT-qPCR)	Inflammation, ECM remodeling, SASP genes	Multi-organ

**Fig. 1:**
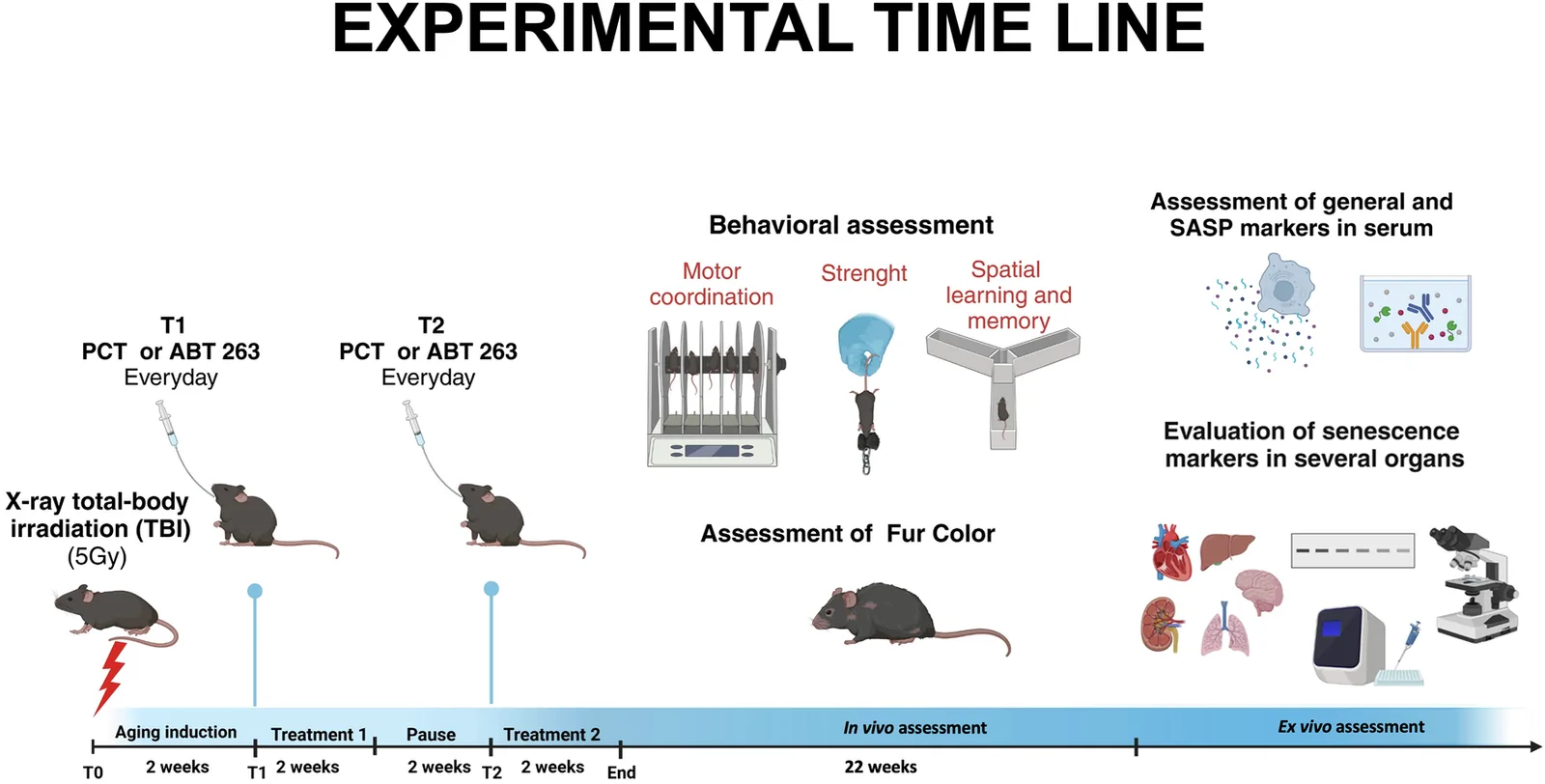
Experimental design and timeline. Animals were divided into four treatment conditions: Untreated controls (no irradiation, no drug); Irradiated-only; Drug-only; Irradiated + drug. Timeline: - Week 0 (T0): X-ray total body irradiation (5 Gy) or sham procedure for controls. - Week 2 (T1): First treatment cycle begins (2 weeks daily oral gavage). - Week 4 (T1 End): First treatment cycle ends; washout period begins. - Week 6 (T2): Second treatment cycle begins (2 weeks daily oral gavage). - Week 8 (T2 End): Second treatment cycle ends; observation period begins. - Week 30 (End Point): Animals sacrificed and comprehensive assessment performed. Behavioral tests (Rotarod, weight-lifting, Y-maze) were conducted at baseline (week 0), pre-treatment (week 4), and endpoint (week 30). Serum biomarkers and tissue analyses were performed at endpoint. Two senotherapeutic compounds were tested: ABT-263 (50 mg/kg, reference senolytic) and piceatannol (PCT, 15 mg/kg, test compound). This resulted in eight experimental groups total (*n* = 6 per group for behavioral tests; *n* = 3 per group for histological and molecular analyses).

These included functional assessments of specific organs and tissues, indirect biochemical markers of physiological function, and analysis of cellular senescence biomarkers. All parameters were measured according to the experimental timeline in Fig. 1.

In line with the experimental design, irradiated animals were compared with untreated controls at 30 weeks post-irradiation. We used a top-down approach: first assessing overall organ and system functionality, then examining tissue-level senescence, and finally performing molecular biomarker analysis.

### Organs and apparatus functionality

**Fig. 2:**
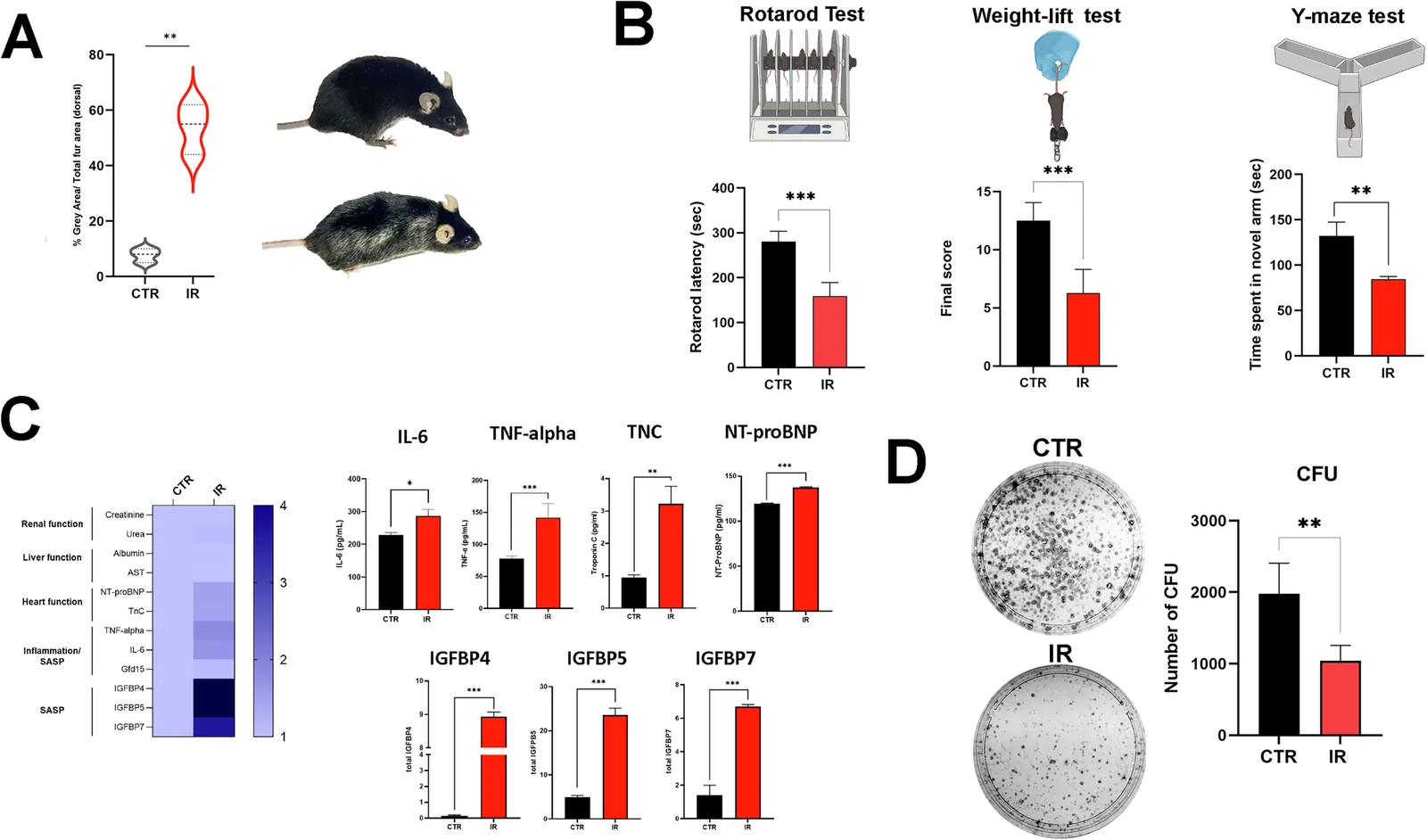
Phenotypic evaluation, behavioral test results, ELIZA, and CFU analysis. **A** Graph showing the percentage of dorsal fur graying relative to the total dorsal fur area in control (CTRL) and irradiated (IR) mice. Representative dorsal fur images from two animals are shown on the right. **B** Histograms displaying the results of the rotarod test (motor coordination), weight-lifting test (strength), and modified Y-maze test (spatial and memory learning) in CTRL and IR mice. **C** Heat map representing the relative abundance of cytokines in plasma samples from CTRL and IR mice. The color scale indicates normalized concentration values, with blue corresponding to higher levels and violet to lower levels, as shown in the scale bar. Data were z-score normalized per row. Histograms on the right depict cytokines showing statistically significant differences between groups. **D** CFU assay for evaluating MSC self-renewal capacity. Representative images of MSC-derived clones from CTRL and IR samples are shown on the right. For all the experiments: *p < 0.05; **p < 0.01; ***p < 0.001. Data represent mean ± SEM from *n* = 3 animals per group. For all statistically significant comparisons, retrospective post-hoc power analysis was performed using G*Power 3.1; the corresponding effect sizes (Cohen’s d) and achieved power values (1-β) are reported in Supplementary File 3.

We began by evaluating phenotypic changes in the fur (Fig. 2A). Irradiated mice developed a dull, grayish, and disheveled coat compared with controls. To assess brain performance, we conducted behavioral tests that provided insights into motor coordination, strength, and spatial learning and memory (Fig. 2B). Irradiated mice showed significant declines in motor coordination, strength, and spatial learning and memory compared to controls.

Kidneys, liver, and heart functionality were assessed by measuring circulating biomarkers: creatinine and urea (renal function), albumin and aspartate aminotransferase [AST] (liver function), and N-terminal pro-B-type natriuretic peptide [NT-proBNP] and troponin C [TnC] (cardiac function) (Fig. 2C). Only a mild increase in NT-proBNP and TnC was observed in irradiated mice, suggesting no overt impairment in the analyzed organs.

Advancing age is characterized by low-grade systemic inflammation (inflammaging), driven by the accumulation of senescent cells and deterioration of immune function (immunosenescence), leading to increased circulating SASP factors [27]. In irradiated mice, we observed a significant increase in TNF-α and IL-6, key pro-inflammatory cytokines, as well as a strong upregulation of IGFBP4, IGFBP5, and IGFBP7, SASP factors that we have identified as playing a causative role in the propagation of senescence [8–10] (Fig. 2C).

Aging is also linked with a reduced self-renewal capacity of tissues and organs due to stem cell exhaustion. In our model, irradiated mice showed a marked reduction in MSC colony-forming unit (CFU) clones (Fig. 2D). The CFU assay, a standard test for stem cell self-renewal capacity, clearly indicated MSC exhaustion [28]. The functional decline of MSCs has profound consequences on organismal homeostasis and tissue renewal, as these cells contribute to osteogenesis, chondrogenesis, and adipogenesis, and secrete trophic factors that support tissue repair and hematopoiesis [28, 29].

### Tissue-level senescence and related phenomena

**Fig. 3:**
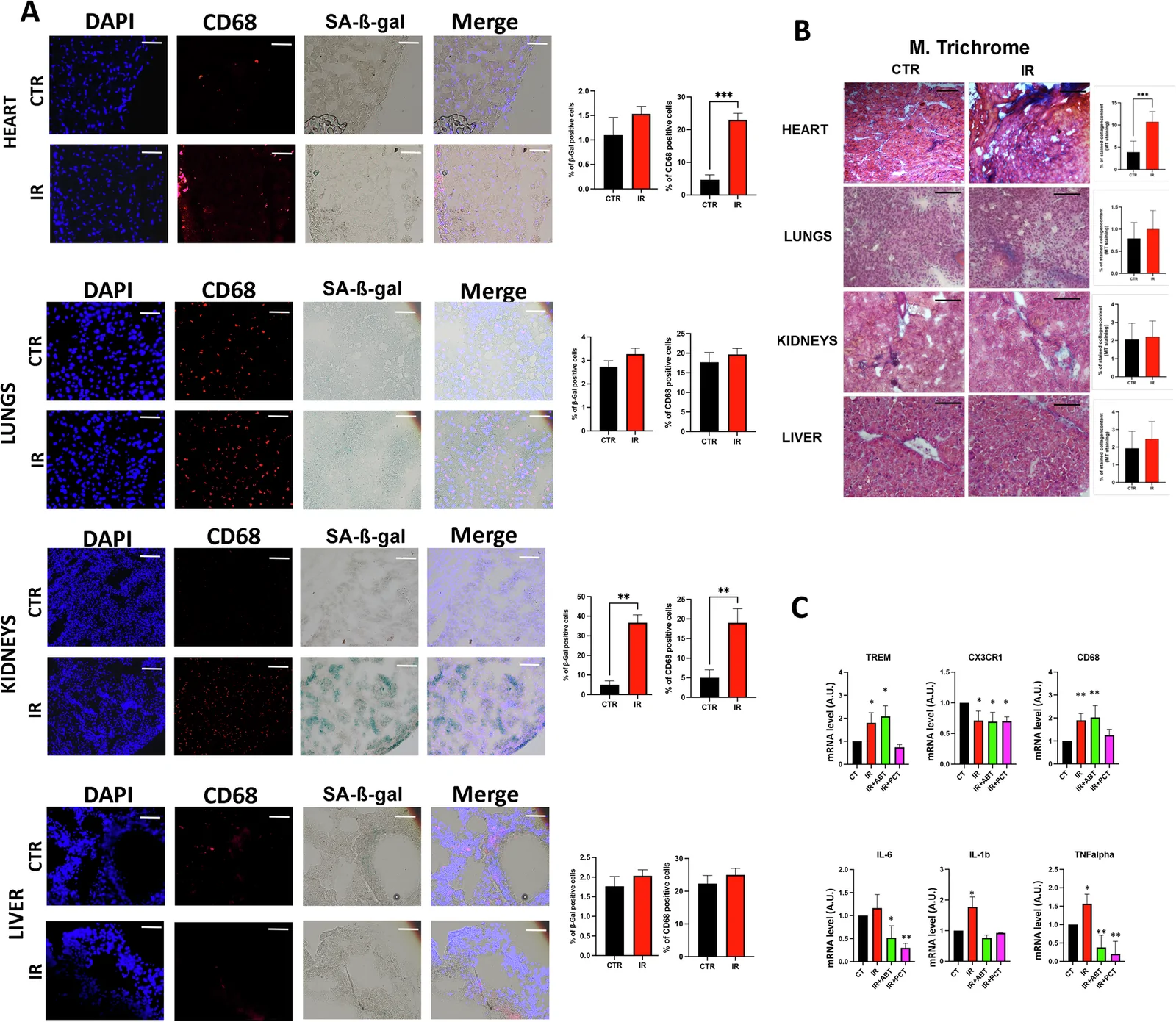
Tissue-level senescence and related phenomena. **A** Representative histological sections and quantification of senescence and macrophage infiltration. Immunofluorescence staining shows CD68+ cells (red), while cell nuclei are counterstained with DAPI (blue). Senescence-associated β-galactosidase (SA-β-gal) activity is indicated by blue histochemical staining. Histograms represent the quantitative analysis of the percentage of SA-β-gal positive (senescent) cells and CD68+ cells within each experimental group. **B** Masson’s trichrome staining in irradiated and control samples. Histograms show the collagen-positive area (blue). Representative tissue sections are shown. **C** Histograms representing the relative abundance of total brain mRNAs in samples from CTRL, IR, IR ABT, and IR PCT groups. For all the experiments: *p < 0.05; **p < 0.01; ***p < 0.001. Data represent mean ± SEM from *n* = 3 animals per group. For histological quantification, at least 5 fields per section and 3 sections per animal were analyzed. For all statistically significant comparisons, retrospective post-hoc power analysis was performed using G*Power 3.1; the corresponding effect sizes (Cohen’s d) and achieved power values (1-β) are reported in Supplementary File 3.

Senescent cells are normally cleared within days to weeks by immune cells. However, as we age, their burden progressively exceeds a critical threshold, leading to their accumulation. This persistent accumulation, driven by proapoptotic and pro-inflammatory SASP factors, may promote tissue damage and contribute to the onset or progression of various age-related diseases [11]. In irradiated mice, the percentage of senescent cells was significantly increased in kidneys (Fig. 3A). The heart showed a trend toward increased area occupied by senescent cells compared with controls, although this difference was not statistically significant (Fig. 3A). Lung tissue showed no apparent increase in senescent cell burden following irradiation, suggesting tissue-specific variability in radiation-induced senescence.

Within tissues, senescent cells form inflamed niches that inhibit stem cell proliferation and tissue regeneration. These areas often contain CD68(+) macrophages and monocytes [30, 31]. We found higher CD68(+) cell abundance in the heart and kidneys of irradiated animals compared to the controls (Fig. 3A).

Senescent cells, via SASP, modify the extracellular matrix (ECM) and promote fibrotic tissue accumulation (fibro-aging), impairing organ function [32, 33]. Masson’s trichrome staining revealed clear fibrosis in the hearts of irradiated mice (Fig. 3B).

Overall, heart and kidney tissues showed the most pronounced senescence and associated pathological changes.

Regarding the brain, the quantitative analysis was more challenging than for peripheral organs due to the brain’s cellular heterogeneity. Unlike peripheral tissues, where macrophages mediate clearance, brain inflammaging is primarily driven by microglia and astrocytes, requiring specialized evaluation approaches [34].

To obtain a preliminary molecular characterization of the neuroinflammatory state induced by peripheral radiation-driven senescence, we analyzed the expression of a panel of microglial activation and neuroinflammatory markers by RT-qPCR in whole-brain tissue lysates from all experimental groups. Despite the deliberate shielding of the head region during irradiation, irradiated mice showed significant upregulation of *Trem2*, *Cd68*, *Il-1β*, *Il-6*, and *Tnfα* compared to controls (Fig. 3C), indicating that peripheral senescence and the associated systemic SASP are sufficient to trigger a neuroinflammatory response in the absence of direct brain irradiation [34]. Consistent with microglial activation, *Cx3cr1*, a marker of homeostatic microglial status that is downregulated upon activation [34], was significantly reduced in irradiated mice, confirming a shift from a quiescent toward an activated microglial phenotype (Fig. 3C).

Taken together, these data support a model in which circulating SASP factors, including IGFBP4, IGFBP5, and IGFBP7, act as endocrine mediators of remote neuroinflammatory activation, extending the senescence cascade from peripheral tissues to the brain. We acknowledge that whole-brain lysate analysis dilutes cell-type-specific signals, and that dedicated region-specific or single-cell resolution approaches will be necessary to fully characterize the spatial and cellular distribution of neuroinflammatory changes in this model.

### Molecular biomarker analysis

Senescence is regulated by a core molecular network including *Rb1*, *Trp53* (P53), *Cdkn1a* (P21), and *Cdkn2a* (P16) [5, 6]. In addition, senescent cells initiate a transcriptional program that activates and/or upregulates immune modulators, extracellular matrix (ECM) remodelers, and SASP components, thereby promoting the spread of senescence (secondary senescence) [35, 36].

**Fig. 4:**
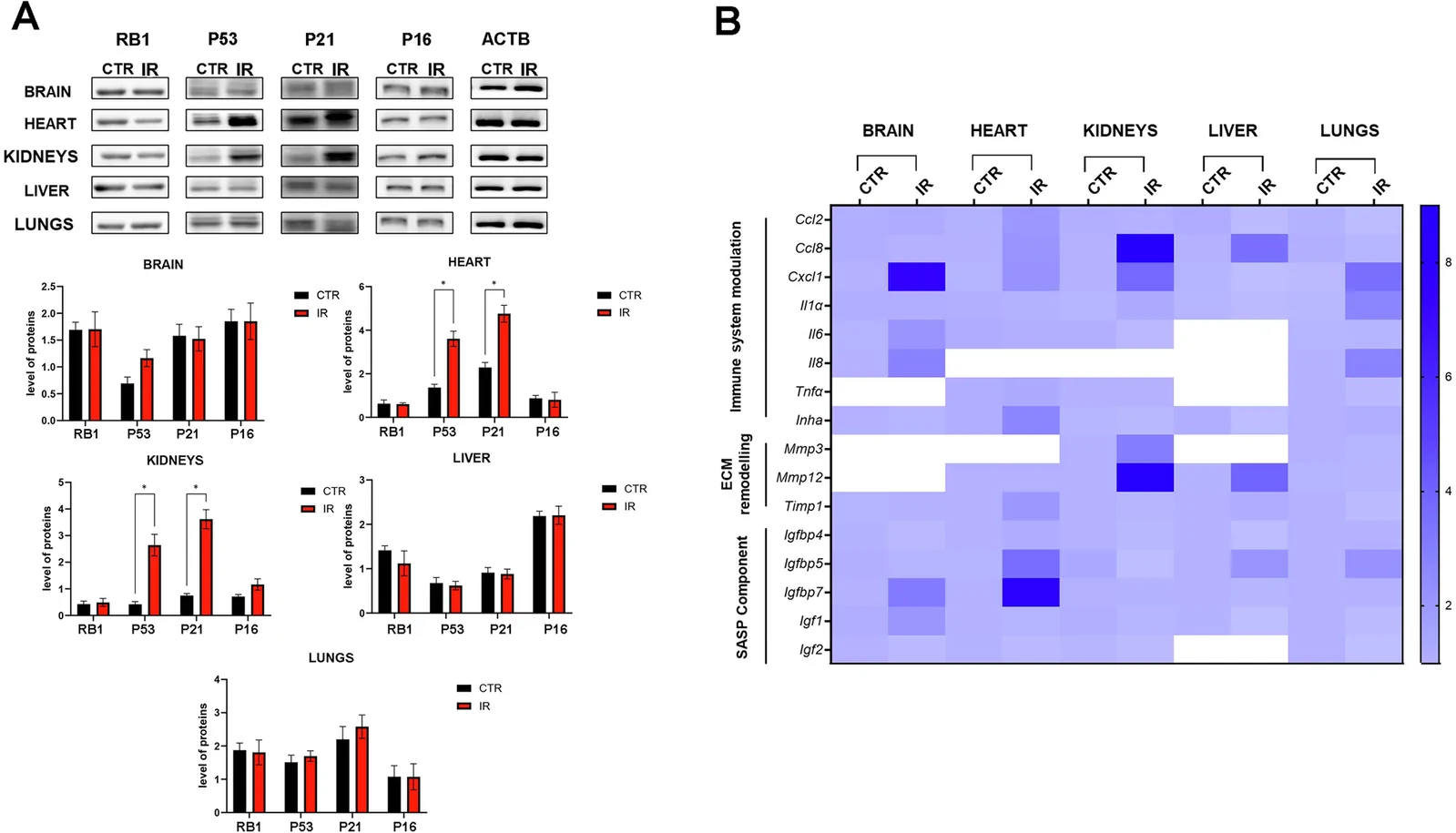
Molecular biomarker analysis. **A** Western blot analysis of the senescence core regulatory network. Representative protein band images are shown. Histograms display protein expression levels normalized to β-actin. **B** Heat map representing the relative abundance of mRNAs in samples from CTRL and IR mice. The color scale indicates normalized values, with blue corresponding to higher levels and violet to lower levels, as shown in the scale bar. Data were z-score normalized per row. For all the experiments: *p < 0.05. Data represent mean ± SEM from *n* = 3 animals per group.

Western blot analysis revealed increased P53 and P21 protein levels in heart and kidney samples from irradiated mice compared with controls (Fig. 4A). No changes were observed for RB1 or P16 across tissues (Fig. 4A). This was expected, as whole tissue lysates were analyzed and senescent cells constitute only a fraction of total cells. The strongest protein changes were in heart and kidney, the tissues with the most evident pathological alterations. The significant changes observed in P53 and P21, which are more broadly expressed senescence markers, better reflect the overall senescence burden in these tissues.

mRNA analysis, also performed on whole tissue lysates, revealed upregulation of genes related to immune modulation, ECM remodeling, and SASP production in multiple tissues (Fig. 4B). This suggests that even a small senescent cell population can influence neighboring cells’ transcriptional programs via SASP.

### Effects of PCT on X-irradiated aged mice

Having established a mild aging model characterized by impaired function in selected organs, the presence of senescent cells and related phenomena, and alterations in key senescence biomarkers, we next investigated the senotherapeutic potential of piceatannol (PCT). For benchmarking, we compared its effects with the well-characterized senotherapeutic agent ABT-263.

While human data on PCT remain limited, resveratrol studies can provide preliminary safety benchmarks. However, PCT’s distinct pharmacokinetic profile, unknown long-term effects, and possible metabolic differences necessitate direct validation [37–39]. To facilitate translation and ensure safety, we selected a low PCT dose based on human-equivalent dose (HED) calculations derived from animal-to-human scaling principles, which account for differences in metabolic rate and physiological turnover between species. The chosen dose falls within the safety range suggested by the only clinical study conducted so far [38]. ABT-263 dosage was based on previously published murine studies [40].

For the evaluation, we focused exclusively on the organ functions, tissues, and molecular biomarkers that showed significant alterations in the irradiated mouse model compared with untreated controls (Supplementary file 1).

### PCT enhanced organ and system function while lowering circulating pro-inflammatory cytokines and SASP factors

**Fig. 5:**
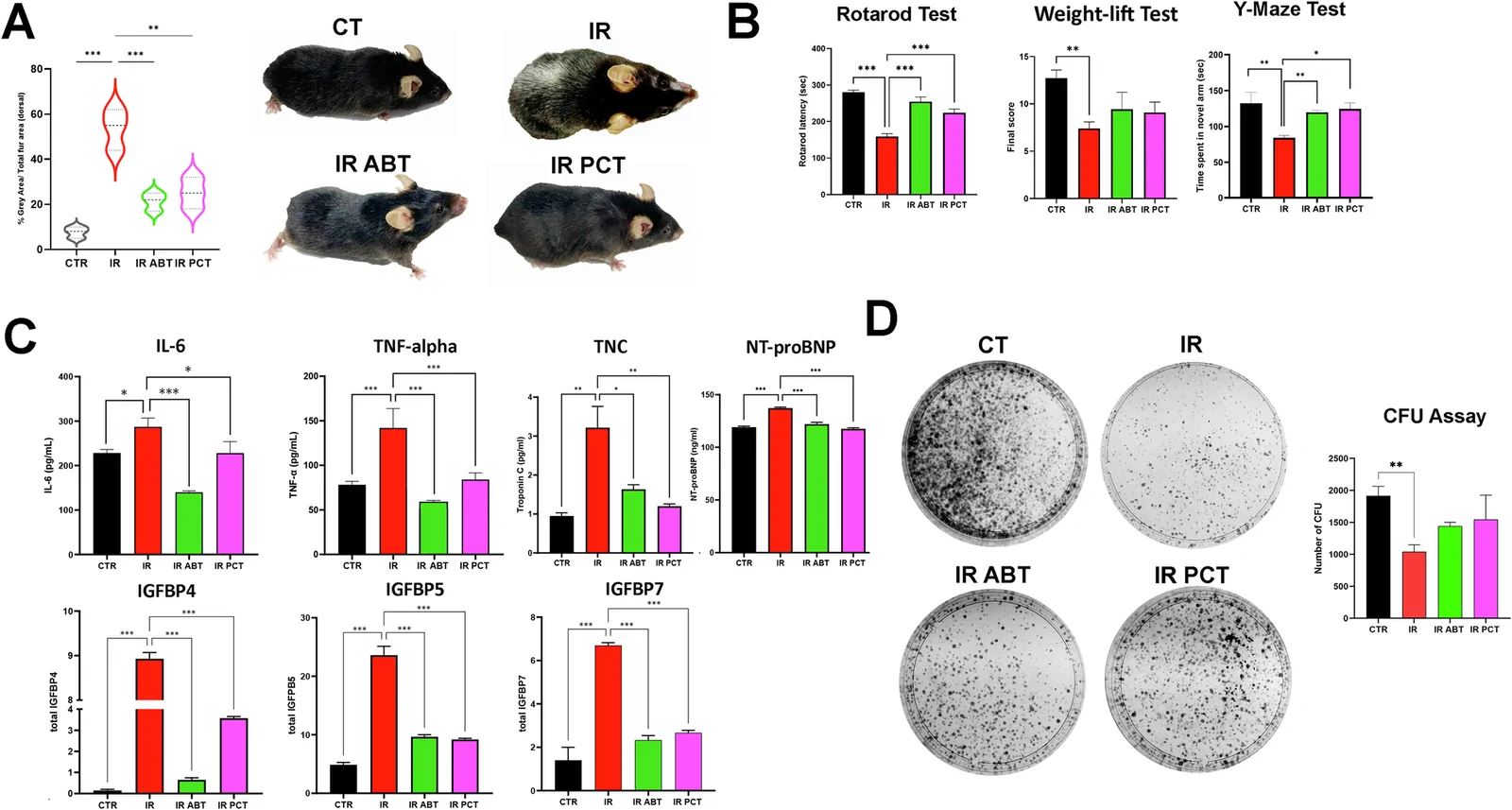
Phenotypic evaluation, behavioral test results, ELISA, and CFU analysis in PCT-treated animals. **A** Graph showing the percentage of dorsal fur graying relative to the total dorsal fur area in control (CTRL), irradiated (IR), irradiated mice treated with ABT-263 (IR ABT), and irradiated mice treated with PCT (IR PCT). Representative dorsal fur images from four animals are shown on the right. **B** Histograms displaying the results of the rotarod test (motor coordination), weight-lifting test (strength), and modified Y-maze test (spatial and memory learning) in the same groups of animals shown in (**A**). **C** Histograms representing the relative abundance of cytokines, SASP factors, and heart biomarkers in plasma samples from CTRL, IR, IR ABT, and IR PCT groups. **D** CFU assay for evaluating MSC self-renewal capacity. Representative images of MSC-derived clones are shown on the right. For all the experiments: *p < 0.05; **p < 0.01; ***p < 0.001. Data represent mean ± SEM from *n* = 3 animals per group. For all statistically significant comparisons, retrospective post-hoc power analysis was performed using G*Power 3.1; the corresponding effect sizes (Cohen’s d) and achieved power values (1-β) are reported in Supplementary File 3.

Treatment with PCT significantly reduced fur changes in irradiated mice (Fig. 5A). PCT also improved motor coordination, spatial learning and memory (Fig. 5B). An increase in muscle strength was observed, though it did not reach statistical significance. PCT lowered circulating pro-inflammatory cytokines (TNF-α, IL-6) and SASP factors (IGFBP4, IGFBP5, IGFBP7), as well as the cardiac stress markers TnC and NT-proBNP (Fig. 5C).

The number of MSC-CFU clones from irradiated mice treated with PCT increased compared with reference animals, although the difference was not statistically significant (Fig. 5D).

### PCT mitigated kidney senescence and associated phenomena

**Fig. 6:**
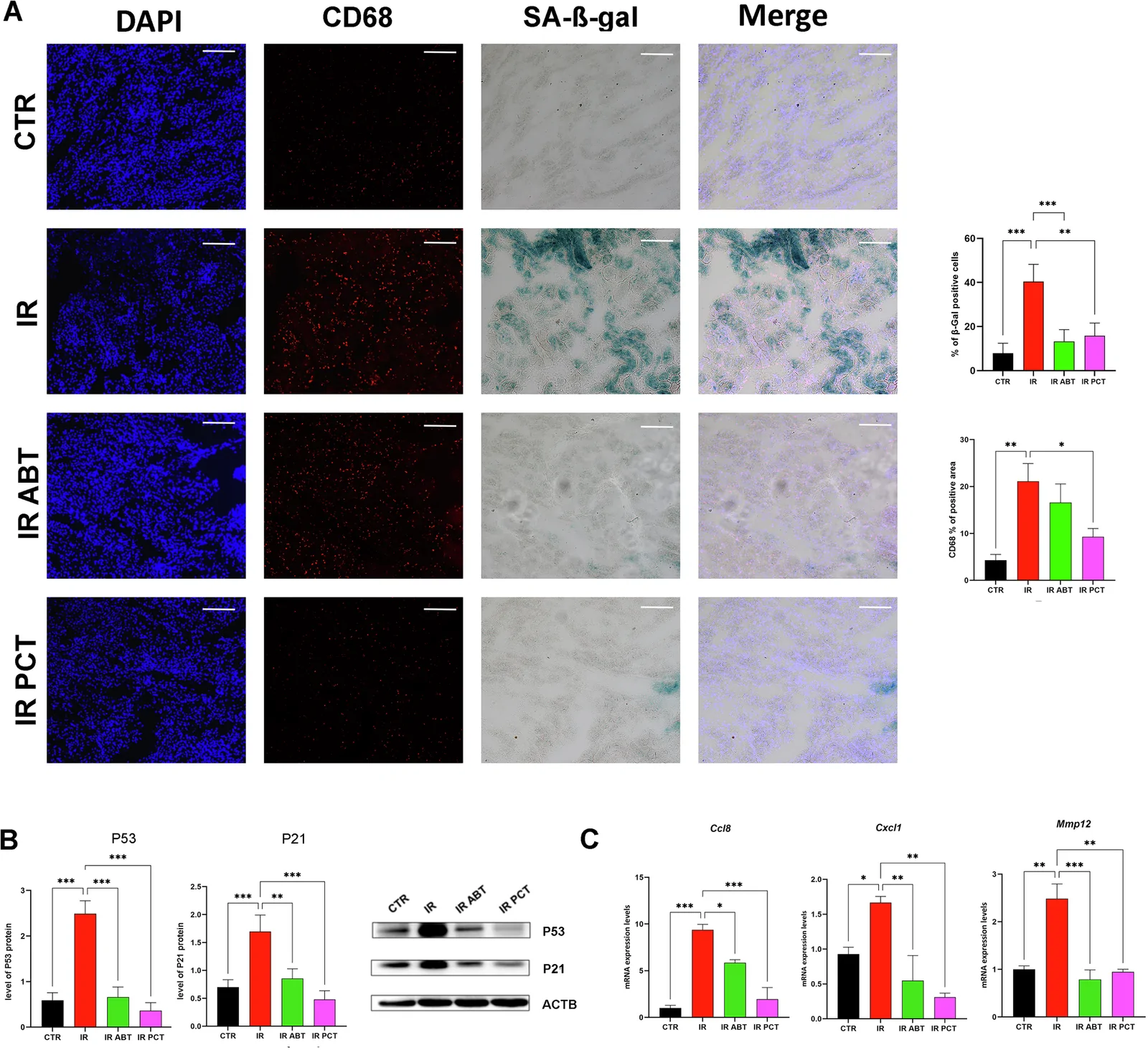
PCT effects on kidney senescence and associated phenomena. **A** Representative histological sections and quantification of senescence and macrophage infiltration. Immunofluorescence staining shows CD68+ cells (red), while cell nuclei are counterstained with DAPI (blue). Senescence-associated β-galactosidase (SA-β-gal) activity is indicated by blue histochemical staining. Histograms represent the quantitative analysis of the percentage of SA-β-gal positive (senescent) cells and CD68+ cells within each experimental group. **B** Western blot analysis of P53 and P21 proteins. Representative protein band images are shown. Histograms display protein expression levels normalized to β-actin. **C** Histograms representing the relative abundance of mRNAs in samples from CTRL, IR, IR ABT, and IR PCT groups. For all the experiments: *p < 0.05; **p < 0.01; ***p < 0.001. Data represent mean ± SEM from *n* = 3 animals per group.

PCT markedly reduced the percentage of senescent cells and CD68(+) foci in the kidney parenchyma of irradiated mice compared with reference animals (Fig. 6A). This was accompanied by a significant decrease in P53 and P21 protein levels, which had been upregulated following irradiation (Fig. 6B). PCT also lowered mRNA expression of immune modulators, ECM remodelers, and SASP components that were elevated in kidney tissue from irradiated mice (Fig. 6C).

### PCT reduced heart senescence and associated phenomena

**Fig. 7:**
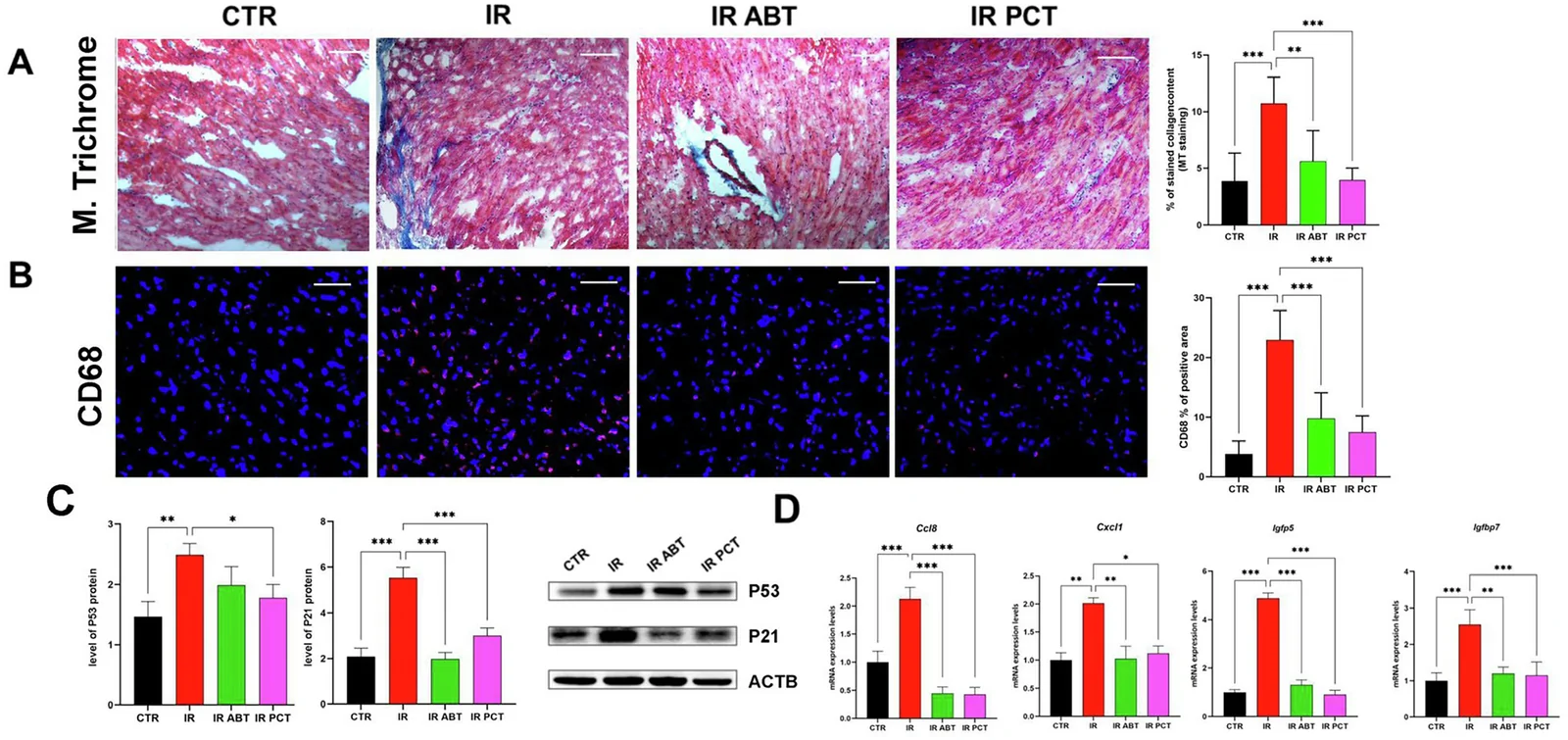
PCT effects on heart senescence and associated phenomena. **A** Masson’s trichrome staining in CTRL, IR, IR ABT, and IR PCT samples. Histograms show the collagen-positive area (blue). Representative tissue sections are shown. **B** Immunofluorescence analysis of CD68(+) cells (red) in the indicated samples. Cell nuclei were stained with DAPI (blue). Histograms show the percentage of CD68(+) cells. Representative tissue sections are shown. **C** Western blot analysis of P53 and P21 proteins. Representative protein band images are shown. Histograms display protein expression levels normalized to β-actin. **D** Histograms representing the relative abundance of mRNAs in samples from CTRL, IR, IR ABT, and IR PCT groups. For all the experiments: *p < 0.05; **p < 0.01; ***p < 0.001. Data represent mean ± SEM from *n* = 3 animals per group. For all statistically significant comparisons, retrospective post-hoc power analysis was performed using G*Power 3.1; the corresponding effect sizes (Cohen’s d) and achieved power values (1-β) are reported in Supplementary File 3.

PCT treatment markedly decreased fibrotic area and CD68(+) cell foci in the hearts of irradiated mice (Fig. 7A, B). This was associated with a significant decrease in P53 and P21 protein expression (Fig. 7C). PCT also reduced mRNA levels of immune modulators and SASP components that were upregulated in heart tissue from irradiated mice (Fig. 7D).

### PCT attenuated peripheral senescence-driven neuroinflammation

Having observed that peripheral radiation-induced senescence triggers a neuroinflammatory response in the shielded brain, evidenced by upregulation of microglial activation markers and pro-inflammatory mediators, we next examined whether PCT treatment was associated with attenuation of this response.

RT-qPCR analysis of whole-brain tissue lysates revealed that PCT-treated irradiated mice showed reduced expression of pro-inflammatory mediators compared to untreated irradiated animals, with Il-6 reaching the highest statistical significance compared with other markers (Fig. 3C). A consistent trend toward reduction was observed for *Trem2*, *Cd68*, *Il-1β*, and *Tnfα*, suggesting a broad attenuation of microglial activation and neuroinflammatory signaling (Fig. 3C). These findings are consistent with PCT’s suppression of circulating IGFBP4, IGFBP5, and IGFBP7 the peripheral SASP factors we have identified as causal drivers of remote organ senescence, thereby interrupting the endocrine cascade that propagates neuroinflammatory activation from peripheral tissues to the brain.

Notably, *Cx3cr1* expression, which was significantly downregulated in irradiated mice reflecting a shift from homeostatic toward activated microglial phenotype, did not recover to control levels following PCT treatment (Fig. 3C). This observation suggests that, while PCT effectively reduces the peripheral senescence signal driving neuroinflammation, full restoration of microglial homeostatic status may require more prolonged treatment or additional interventions targeting the CNS compartment directly. This question will be addressed in future studies employing region-specific and single-cell resolution approaches.

### PCT and ABT-263 effects on healthy mice

Non-irradiated mice treated with either PCT or ABT-263 were included to evaluate potential adverse effects in young adults. These animals were 4 weeks old at the start of the experiment, and analyses were performed 30 weeks later as part of the experimental time course.

At this age, aging-related changes are not expected; thus, senotherapeutic effects could not be detected. Functional and molecular analyses revealed no alterations suggestive of adverse effects, confirming the safety of both compounds under the tested conditions (data not shown).

## Discussion

### Usefulness of our X-ray–irradiated mice model

The use of X-ray-irradiated mice as an aging model provides a more consistent starting point than relying on chronological aging, which is highly variable between individuals. By inducing mild aging through a single sublethal irradiation, we simulated early aging phenotypes in the absence of severe organ dysfunction. This model is particularly valuable for evaluating anti-aging therapies aimed at prevention, rather than reversal, of advanced functional decline.

Unlike naturally aged cohorts, which display wide heterogeneity in biological age, irradiation generates a synchronized onset of senescence-associated features. This allows for more reproducible longitudinal studies and facilitates the detection of subtle therapeutic effects.

The head region was intentionally protected during X-ray exposure to avoid acute radiation damage to the brain while permitting systemic induction of aging-related changes. Our goal was to generate a *mild aging* phenotype rather than acute radiation toxicity. In this context, the behavioral deficits observed in irradiated mice, including impaired motor coordination and spatial memory, are most plausibly driven by peripheral aging mechanisms rather than direct brain damage.

Importantly, our model reflects a stage comparable to healthy middle-aged humans who retain functional organ reserve but already show increased senescent cell burden, senescence-associated tissue remodeling, and low-grade inflammation. Indeed, the “threshold theory of senescent cell accumulation” suggests that when the density of senescent cells surpasses a specific limit, the SASP-driven expansion of senescence via paracrine and endocrine pathways begins to outstrip the immune system’s clearative capacity. Furthermore, the rising concentration of SASP-associated factors may actively suppress immune efficiency, creating a feedback loop that accelerates the buildup of these cells [11].

However, several critical limitations must be acknowledged. Irradiation induces acute, synchronized senescence, whereas natural aging involves gradual, heterogeneous accumulation of senescent cells over decades. Radiation-induced DNA damage may trigger distinct cellular responses compared to the multifactorial stressors of physiological aging (telomere attrition, mitochondrial dysfunction, oxidative stress, etc.). The pattern of tissue damage following acute irradiation may differ from the progressive, stochastic deterioration characteristic of natural aging. In our model only male mice were studied; moreover, sex is a major factor in aging biology and PCT effects may be sex-dependent. Young adult mice (8 weeks) were used; responses to intervention may differ in middle-aged or geriatric animals. We performed a short observation period (30 weeks total) and long-term effects of PCT treatment remain unknown.

Despite these limitations, the controlled nature of this model enabled us to demonstrate proof-of-concept for PCT’s senotherapeutic potential. The characteristics observed‚ the senescent cell accumulation, inflammatory milieu, and functional deficits without overt organ failure make it suitable for testing interventions designed to maintain health span and prevent progression to irreversible dysfunction.

### PCT as a promising senotherapeutic

While resveratrol has shown anti-aging activity in multiple models, its clinical translation is constrained by poor bioavailability and a short plasma half-life, requiring gram-scale daily dosing in humans to achieve modest systemic exposure [41, 42]. In contrast, piceatannol (PCT) displays a more favorable pharmacokinetic profile, enabling biological activity at substantially lower and more translatable doses. In our study, the administered PCT dose (15 mg/kg in mice, corresponding to ~1.2 mg/kg in humans) falls within a range realistically achievable by supplementation, unlike the supraphysiological doses typically required for resveratrol. Direct comparison with resveratrol is complicated by major pharmacokinetic and metabolic differences, including the partial in vivo conversion of resveratrol into PCT and the lack of dose-equivalent systemic exposure across compounds. Accordingly, the primary goal of this work was to establish proof-of-concept for PCT.

Given the limited number of human studies on PCT, resveratrol data provide a cautious reference for safety, although their applicability is restricted by PCT’s distinct metabolism and pharmacokinetics. On this basis, we selected a low PCT dose within the safety range suggested by the only available clinical data and by resveratrol reference studies. Notably, our experimental approach involves the use of safe, nutritionally derived senolytics and senomorphics. Together with previous work on “anti-senescence foods,” our findings support the development of nutraceutical strategies for healthier aging, positioning PCT as a promising candidate for senotherapeutic intervention [17, 43].

### Effects on inflammation and systemic markers

Consistent with this favorable pharmacological profile, PCT treatment produced broad functional improvements across multiple organ systems in our model.

PCT treatment significantly enhanced performance in the rotarod test, indicating improved motor coordination and balance, and increased exploration time in the novel arm of the Y-maze, suggesting preserved spatial working memory. While these behavioral improvements are encouraging, comprehensive evaluation of cognitive functions‚ including long-term memory, complex learning tasks, and executive functions‚ will be necessary to fully characterize PCT’s neuroprotective effects in aging.

The immune system plays a crucial role in the body’s defense mechanisms. As individuals age, the immune system becomes chronically active, as evidenced by elevated levels of inflammatory markers and immune cell activation in both the bloodstream and tissues, a phenomenon known as “inflammaging” [17, 31]. Persistent low-grade inflammation in the elderly contributes to the development of many aging-related diseases, including cancer. Thus, the reduction of inflammatory cytokines (TNF-α, IL-6) and SASP factors (IGFBP4, IGFBP5, IGFBP7) by PCT is a critical outcome for promoting healthy aging. By lowering circulating and tissue levels of IGFBP4/5/7, PCT specifically targets this validated senescence-propagation network, providing a mechanistically coherent explanation for the observed reductions in senescent burden, inflammation, and functional decline.

Our findings reveal tissue-specific variability in both senescence accumulation and PCT responsiveness following radiation exposure. Among the organs examined, kidney and heart showed the most pronounced senescence-associated alterations, including increased SA-β-gal positive cells, elevated CD68+ inflammatory foci, and upregulation of senescence markers (P53, P21). The strong effects in kidney and heart are particularly relevant, as cardiovascular and renal dysfunction are leading causes of morbidity and mortality in aging populations. The preservation or improvement of function in these critical organs, even in the absence of uniform effects across all tissues, has substantial implications for health-span.

The tissue-specific nature of PCT effects could result from (i) differential bioavailability: variation in PCT uptake or metabolism across tissues; (ii) cell-type specific sensitivity: different cell types may respond variably to PCT; (iii) baseline senescent cell burden: tissues with higher initial senescence may show more dramatic improvements; (iv) distinct senescence drivers: radiation may induce senescence through tissue-specific mechanisms.

The observation that senescent cell accumulation preceded measurable organ dysfunction in our model supports the concept of a “tipping point” in senescence burden. In kidneys, for example, we detected increased senescent cells and inflammatory markers even though serum creatinine and urea remained within normal ranges (Fig. 2C). PCT’s ability to reduce senescent cell burden at this preclinical stage suggests potential for preventive intervention before irreversible organ damage occurs.

### Mechanistic insights from integrated in vitro and in vivo findings: evidence for a senomorphic mode of action of PCT

A primary and direct line of evidence supporting the senomorphic classification of PCT derives from the dose-response relationship between the administered dose and the known biological activity of the compound. In the present study, PCT was administered at 15 mg/kg in mice, a dose selected on the basis of human-equivalent dose (HED) calculations corresponding to approximately 1.2 mg/kg in humans.

In our previously published in vitro work [23], we demonstrated that PCT at concentrations consistent with those estimated to be achievable in vivo at this dose does not induce apoptosis in senescent MSC, assessed by Annexin V within 24–48 h of exposure, the standard operational criterion for senolytic activity. In contrast, PCT at these concentrations effectively suppressed SASP secretion and reduced senescence-associated markers, consistent with a senomorphic mechanism.

This pharmacological evidence establishes a dose-dependent mechanistic boundary: at the concentrations used in the present study, PCT operates below the threshold required for senolytic activity and within the range associated with senomorphic effects. The classification of PCT as a senomorphic agent in our experimental system is therefore not inferred from phenotypic data alone, but is grounded in direct experimental evidence at the relevant concentration range.

A further mechanistic argument, grounded in our own published data, directly supports the senomorphic interpretation. Ionizing radiation induces an acute DNA damage response in tissues, resulting in two parallel outcomes: immediate apoptotic death of a fraction of cells, and stable senescence induction in surviving cells (primary senescent cells). This primary senescent population then secretes SASP factors that propagate senescence to neighboring healthy cells, process of secondary senescence that is responsible for the progressive amplification of senescent cell burden over time.

Critically, we have demonstrated in three independent studies that IGFBP4, IGFBP5, and IGFBP7, all components of the SASP detected in our irradiated model, are not merely correlative markers but causal inducers of senescence: administration of each IGFBP individually to healthy cells is sufficient to induce a full senescent phenotype [8–10]. These IGFBPs operate through distinct but convergent mechanisms, ROS–prostaglandin–EP2 signaling (IGFBP4), nuclear retinoic acid receptor activation (IGFBP5), and disruption of insulin/IGF-1/activin signaling (IGFBP7), forming a coordinated network that amplifies and sustains senescence propagation across tissues.

PCT markedly reduces circulating and tissue levels of all three IGFBPs (Figs. 5C, 6C and 7D). By suppressing this causally validated senescence-propagating network, PCT interrupts the feed-forward amplification of secondary senescence. The observed reduction in total SA-β-gal+ cell burden therefore reflects the prevention of secondary senescence accumulation, not the elimination of radiation-induced primary senescent cells. This mechanistic interpretation is fully consistent with a senomorphic mode of action and does not require invoking senolytic activity.

It is also instructive to consider the long-term effects of ABT-263 within this framework. ABT-263 is a well-characterized senolytic that clears senescent cells through BCL-2/BCL-XL-dependent apoptosis induction. Its sustained efficacy at the 30-week endpoint is mechanistically coherent: by eliminating the primary senescent cell population in the early post-irradiation period, ABT-263 removes the principal source of SASP, thereby secondarily attenuating the propagation of senescence to neighboring healthy cells over the subsequent months. Senolysis of primary cells and senomorphic suppression of secondary senescence thus represent two mechanistically distinct but functionally convergent strategies, both capable of reducing total senescent cell burden at a long-term endpoint.

This convergence explains why PCT and ABT-263 produce comparable reductions in SA-β-gal+ cell counts at 30 weeks despite their different primary mechanisms. Importantly, it also underscores that equivalent endpoint phenotypes do not imply equivalent mechanisms, a principle that is well established in the senotherapy literature [15]. The mechanistic distinction between PCT and ABT-263 is supported by their divergent molecular profiles: unlike ABT-263, PCT does not induce apoptosis in senescent cells at physiologically relevant concentrations [23], but specifically suppresses the IGFBP4/5/7-mediated senescence propagation network [8–10], providing a causally grounded mechanistic basis for its classification as a senomorphic agent.

### PCT as a natural tool for promoting healthy aging?

Our experimental approach was based on low-dose PCT treatment before the onset of any detectable and overt signs of organismal aging. The chosen treatment prevented and/or delayed the appearance of inflammation, the increase in circulating SASP factors, the presence of senescent cells in tissues, cognitive decline, and fur graying. These results may pave the way for treating healthy middle-aged individuals with low and safe PCT supplementation to promote a healthy aging process.

### Study limitations and future perspectives

There are intrinsic limits to our experimental approach. Our model represents early-stage, radiation-accelerated aging. Whether PCT would demonstrate similar tissue selectivity in natural aging, where senescence accumulates gradually over decades, remains to be determined.

Some limits of our model could be addressed in future studies, while others face technical and ethical obstacles. The X-ray–irradiated mouse model reduces variability but only partially mimics the natural aging process. We employed a simplified experimental model, a common approach in research to capture the core aspects of a system while retaining explanatory and predictive power. The so-called “minimum adequate model” seeks to reduce variables without diminishing the model’s validity. Once significant outcomes are obtained with this minimal model, further experiments should include additional variables. Our future goal is to analyze the effects of PCT in naturally aged mice, using a broader range of tissue and organ functionality markers beyond those already assessed.

Regarding the brain analyses, the use of whole-brain lysates for RT-qPCR necessarily dilutes cell-type-specific signals and precludes spatial resolution of neuroinflammatory changes. Future studies will employ region-specific dissection, single-cell RNA sequencing, and dedicated microglial isolation protocols to characterize the cellular basis of PCT’s effects on CNS inflammaging with greater precision.

## Conclusion

### Next steps

Having established the potential of PCT as a senotherapeutic in a simplified aging model, we propose a four-phase investigation: (1) validation in healthy middle-aged mice to confirm its anti-aging properties; (2) evaluation in geriatric mice with age-related comorbidities (e.g., cataracts, frailty spectrum disorders, and multi-organ dysfunction); (3) dosage optimization studies; and (4) analysis of additional functions and parameters that decline with age but were not assessed in the present study. While senotherapeutics in aged organisms typically employ senolytic strategies to clear established senescent cell populations via apoptotic pathways, polyphenols exhibit dose-dependent pleiotropism, demonstrating senomorphic activity at lower concentrations and potential senolytic effects at higher doses. This biphasic response warrants systematic evaluation of PCT across a therapeutic range, including the previously tested low dosage and additional higher concentrations.

Our findings position piceatannol as a mechanistically validated senotherapeutic candidate. PCT specifically reduces IGFBP4, IGFBP5, and IGFBP7. By blocking these molecularly defined targets, PCT interrupts the self-amplifying senescence cascade, explaining its multi-organ benefits. This mechanistic coherence, combined with PCT’s favorable pharmacokinetic properties and preliminary safety data in humans, provides strong rationale for advancing PCT to clinical trials with IGFBP levels serving as translational biomarkers for target engagement and therapeutic efficacy.

## Materials and methods

### Animals

C57BL/6 J inbred wild-type male mice were obtained from ENVIGO (IN, USA). Animals (8 week-old) were randomly assigned to eight experimental groups (*n* = 6 per group): 1. Untreated controls (no irradiation, no treatment); 2. Irradiated only (5 Gy X-ray, vehicle treatment; 3. Irradiated + ABT-263; 4. Irradiated + PCT; 5. ABT-263 only (no irradiation); 6. PCT only (no irradiation); 7. Vehicle-treated controls (for drug-only groups); 8. Age-matched baseline controls.

This resulted in a total of 48 mice. All procedures were performed in accordance with protocols approved by the Animal Care and Use Committee of the University of Campania “Luigi Vanvitelli.” Mice were maintained in a specific pathogen-free facility under a 12 h light/dark cycle at 23–24 °C, with free access to standard chow and water, and housed three per cage.

For behavioral assessments and serum biomarker analysis, all six animals per group were tested. For histological and molecular analyses (immunostaining, Western blot, qRT-PCR), tissues from three randomly selected animals per group were used to reduce animal sacrifice while maintaining statistical power for detecting significant differences based on preliminary data and published effect sizes.

### Mice total body irradiation (TBI)

To induce acute aging, 8-week-old male mice were subjected to a sublethal dose of 5 Gy X-ray total body irradiation (TBI) using a Elektra Infinity instrument (Sweden) operating at 6 MeV, under room temperature. The head region was deliberately shielded during exposure to prevent severe acute radiation injury to the brain while allowing systemic induction of aging-associated changes. This design models a condition of preserved brain structure but functional decline driven by peripheral factors such as inflammaging, circulating SASP factors, and reduced trophic support. Mice were allowed to recover for two weeks before randomization into control and treatment groups.

### Treatment and sample collection

Animals were allowed to recover for two weeks following irradiation before treatment initiation. Starting at week 2 post-irradiation (designated as T1), mice received daily oral gavage with either ABT-263 (50 mg/kg, ab218114, ABCAM, MS, USA), PCT (15 mg/kg; P0453, Sigma-Aldrich, MO, USA), or vehicle (saline). The treatment regimen consisted of two cycles:

First cycle: 2 weeks of daily treatment (weeks 2–4 post-irradiation); Washout period: 2 weeks (weeks 4–6 post-irradiation); Second cycle: 2 weeks of daily treatment (weeks 6-8 post-irradiation).

Animals were then monitored without further intervention for an additional 22 weeks. At 30 weeks post-irradiation, mice were sacrificed and blood and organs (heart, kidney, liver, lung, and brain) were collected. Tissues were embedded in OCT and stored at −80 °C or directly stored at −80 °C for subsequent RNA and protein analyses. In parallel, femurs and tibias were freshly harvested for bone marrow extraction and MSC isolation to perform CFU assays.

### Behavioral evaluation

Behavioral tests were conducted to assess motor coordination, muscle strength, and spatial working memory. Mice underwent Rotarod, weight-lifting, and Y-maze tests at baseline and at specific time points during the experimental timeline. Testing sessions were scheduled on separate days to avoid overlap and potential carry-over effects between tasks.

### Rotarod test

Motor coordination and balance were assessed using a Rotarod apparatus (Ugo Basile, Italy). Mice were trained for three consecutive days at 4, 6, and 8 rpm for 200 s per day. On the test day, after a 30 s adaptation period, rotation speed accelerated from 5 to 40 rpm over 5 min. Each mouse underwent three trials separated by 1 h. The latency to fall (s) was automatically recorded, and the mean latency from three or four trials was used for analysis.

### Weight-lifting

Muscle strength was evaluated using a standardized weight-lifting test. The apparatus consisted of a stainless-steel wire ball attached to linked chain segments (13 g each), providing weights from approximately 17 g to 69 g. Mice were lowered by the tail to grasp the wire ball and lifted until the weight was held for up to 3 s. Each weight was attempted up to three times. If successful, heavier weights were tested sequentially. The final score was calculated as the number of links in the heaviest chain held for 3 s × time held, with intermediate scores assigned when duration was <3 s, as previously described [44].

### Y-maze

Short-term spatial memory was assessed using a modified Y-maze with three enclosed arms (30 × 5 × 15 cm) converging at a central triangle (5 × 5 × 5 cm) according to previously reported methods [45]. After three days of handling, mice performed a 5 min sample trial (T1) exploring two arms, while the third arm was blocked. After a 30 min intertrial interval, a retrieval trial (T2) was conducted with all arms open. Latency to enter and time spent in the novel arm were recorded.

### Assessment of Fur color changes

Age-related changes in fur pigmentation were quantified. High-resolution images of the dorsal coat were captured, and the percentage of gray area relative to total fur area was determined using ImageJ software (NIH, MD, USA) with color threshold function (saturation <50, brightness 50–200). At least three images per animal were analyzed, and the mean value was used for analysis.

### Colony-forming unit (CFU) assay

Bone marrow–derived MSCs were isolated from mouse femurs and tibias, plated in α-MEM (Thermo Fisher Scientific, MA, USA) with 15% ES serum (Euroclone, Italy), and cultured at 37 °C in 5% CO₂. Non-adherent cells were removed after 48 h, and adherent cells were expanded to confluence (P0) before trypsinization and subculture (P1). For the CFU assay, 1 × 10³ MSCs were seeded in 60-mm dishes and cultured for 15 days, then fixed and stained with 0.5% crystal violet. After staining, colony images were acquired using the Azure Biosystems 600 Imaging System (Azure Biosystems, CA, USA), and colonies containing ≥50 cells were quantified using ImageJ software (NIH, USA). The procedure was performed as previously described [9].

### Serum collection and analysis

Blood samples were collected by cardiac puncture under terminal anesthesia. Samples were allowed to clot at room temperature, centrifuged at 2000 × *g* for 10 min at 4 °C, and serum was stored at −80 °C. Serum levels of metabolic and inflammatory markers were quantified using ELISA kits or metabolic assay kits, following manufacturer instructions. Protein concentrations were determined via Bradford assay (Bio-Rad, CA, USA). In addition, Western blot analysis for IGFBP4, IGFBP5, and IGFBP7 was performed on serum samples, following the procedures described in the corresponding section below. (Supplementary file 4 contains reagent sources).

### Histology and immunostaining

Tissues were embedded in OCT and stored at −80 °C until processing. Senescence-associated β-galactosidase (SA-β-gal) activity was assessed as a marker of cellular senescence, as previously described [46]. Briefly, sections were cut using a cryostat, washed twice in ice-cold PBS (Microgem, Italy), and fixed on ice for 10 min in 2% formaldehyde/0.2% glutaraldehyde (Sigma Aldrich, MO, USA) in PBS. Slides were incubated overnight at 37 °C in SA-β-gal staining solution (pH 6.0), washed, counterstained with Mayer’s hematoxylin (Sigma Aldrich, MO, USA), dehydrated through graded ethanol (Sigma Aldrich, MO, USA), cleared in xylene (Sigma Aldrich, MO, USA), and mounted.

Masson’s trichrome staining was performed on frozen OCT sections using the MTS Kit (Cat. HT15-KT, Sigma-Aldrich, MO, USA), according to a previously published protocol for frozen sections [47]. Sections were fixed in 4% paraformaldehyde (Sigma Aldrich, MO, USA) for 10 min at room temperature before staining.

For immunofluorescence, OCT sections were fixed in 4% paraformaldehyde for 10 min, washed in PBS, permeabilized with 0.3% Triton X-100 (Sigma Aldrich, MO, USA) for 10 min, and blocked with 5% ES serum in PBS for 1 h at room temperature. Sections were incubated overnight at 4 °C with primary antibodies against CD68 (see Reagents), followed by fluorescent-labeled secondary antibodies for 1 h at room temperature. Nuclei were counterstained with DAPI (Sigma Aldrich, MO, USA). All images were acquired using a Zeiss Axioscope 5 microscope (Carl Zeiss, Germany) and subsequently analyzed using ImageJ software (NIH, MD, USA). (Supplementary file 4 contains reagent sources).

### Protein extraction and western blotting analysis

Tissues (heart, kidney, liver, lung, and brain) were homogenized in protein extraction buffer containing 0.1% Triton (Bio-Rad, CA, USA) supplemented with protease and phosphatase inhibitor cocktails (Sigma-Aldrich, MO, USA) using a TissueRuptor homogenizer (Qiagen, Germany). Lysates were incubated on ice for 30 min and centrifuged at maximum speed for 15 min at 4 °C. The supernatants were collected, and protein concentrations were determined by Bradford assay. Equal amounts of protein (20 µg) were separated by SDS-PAGE and transferred onto PVDF membranes. Membranes were incubated with primary antibodies (P53, P21, P16, RB1) and HRP-conjugated secondary antibodies. Immunoreactive bands were visualized using ECL Radiance ECL (Azure Biosystems, CA, USA) and acquired with an Azure Biosystems 600 Imaging System (Azure Biosystems, CA, USA). Densitometric quantification was performed using Quantity One software (Bio-Rad, CA, USA). The western blot raw data are in supplementary file 2. (Supplementary file 4 contains reagent sources).

### RNA extraction and quantitative RT-PCR

Total RNA was isolated from tissues using the RNeasy Plus Universal Mini Kit (Qiagen, Germany) and homogenized with the TissueRuptor. RNA purity and concentration were assessed using a NanoDrop One spectrophotometer (Thermo Fisher Scientific, MA, USA). Complementary DNA (cDNA) was synthesized with the ALL-IN-ONE RT MasterMix (Applied Biological Materials, Canada). Quantitative real-time PCR (qPCR) was performed using BrightGreen 2X qPCR MasterMix (Applied Biological Materials, Canada) on the LineGene 9600 real-time PCR system (Bioer Technology, China). Primer sequences are provided in Supplementary Table S4. GAPDH was used as the internal control, and relative gene expression levels were calculated using the 2^ − ΔΔCT method. (Supplementary file 4 contains reagent sources).

### Statistical analysis

Statistical significance was assessed using Student’s *t* test for comparisons between two groups, and ANOVA for comparisons involving more than two groups. Mixed model analysis of variance was used for data with continuous outcomes. All data were analyzed using the GraphPad Prism version 9 statistical software package (GraphPad, CA, USA). Retrospective post-hoc power analysis was performed using G*Power 3.1.9 (Heinrich Heine University Düsseldorf, Germany) to assess the statistical adequacy of the sample sizes employed. Achieved power values (1-β) for all primary outcome variables are reported in the respective figure legends.

## Supplementary information

Supplementary file 1

Supplementary file 2

Supplementary file 3

Supplementary file 4

## Data Availability

The data that support the findings of this study are available from the corresponding authors upon reasonable request.
