# Supplementary material for: Piceatannol as a safe senotherapeutic: ameliorating senescence-associated markers in a radiation-induced murine model
Source: Cell Death Discov. 2026 Jul 14;12:408. doi: 10.1038/s41420-026-03253-1 (PMC13646293; doi:10.1038/s41420-026-03253-1)

Sup File

Raw data Western blot

Figure 4

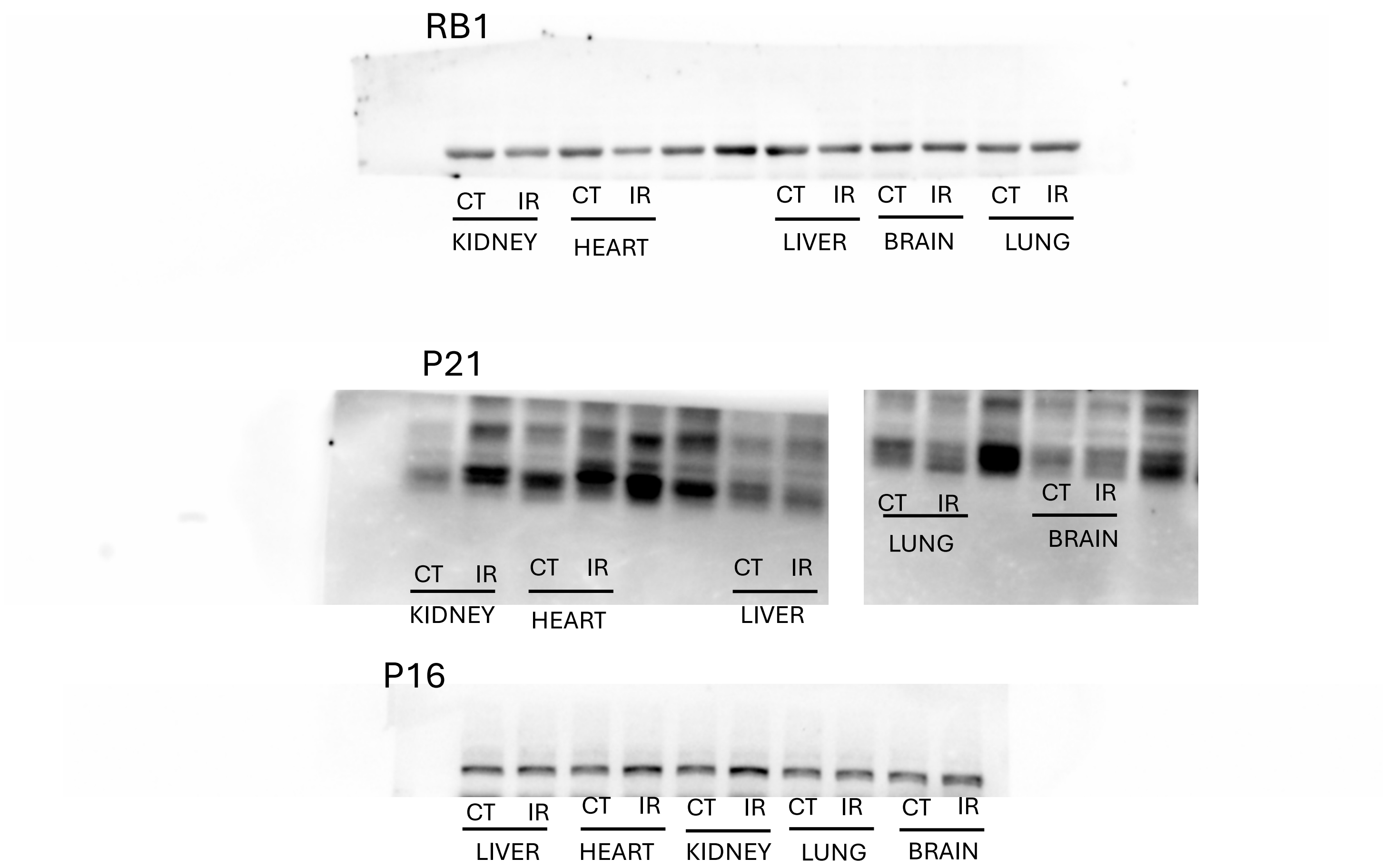
RB1
CT
IR
KIDNEY
CT
IR
HEART
CT
IR
LIVER
CT
IR
BRAIN
CT
IR
LUNG
P21
CT
IR
KIDNEY
CT
IR
HEART
CT
IR
LIVER
CT
IR
LUNG
CT
IR
BRAIN
P16
CT
IR
LIVER
CT
IR
HEART
CT
IR
KIDNEY
CT
IR
LUNG
CT
IR
BRAIN

P53

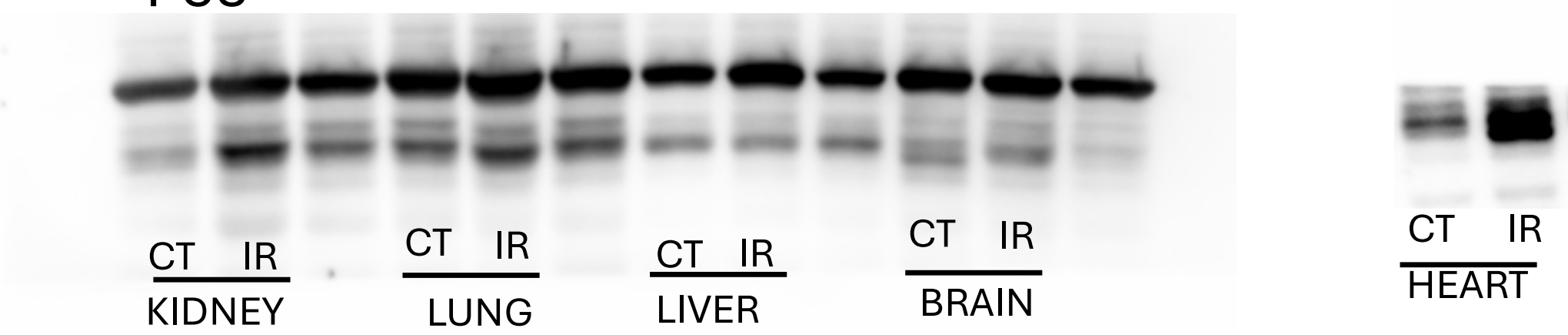
CT IR
KIDNEY
CT IR
LUNG
CT IR
LIVER
CT IR
BRAIN
CT IR
HEART

b-ACTIN

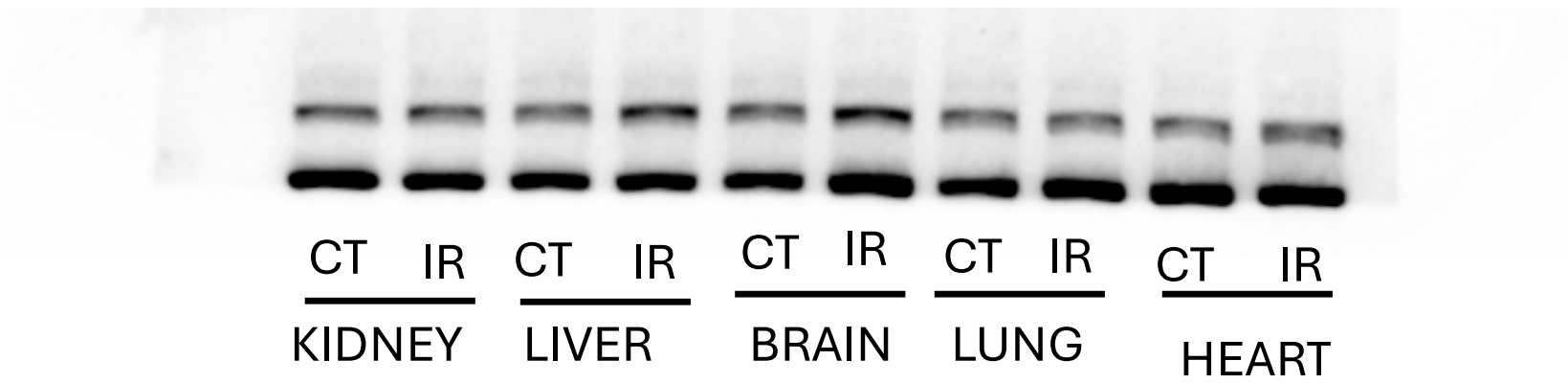
CT IR
KIDNEY
CT IR
LIVER
CT IR
BRAIN
CT IR
LUNG
CT IR
HEART

Figure 6

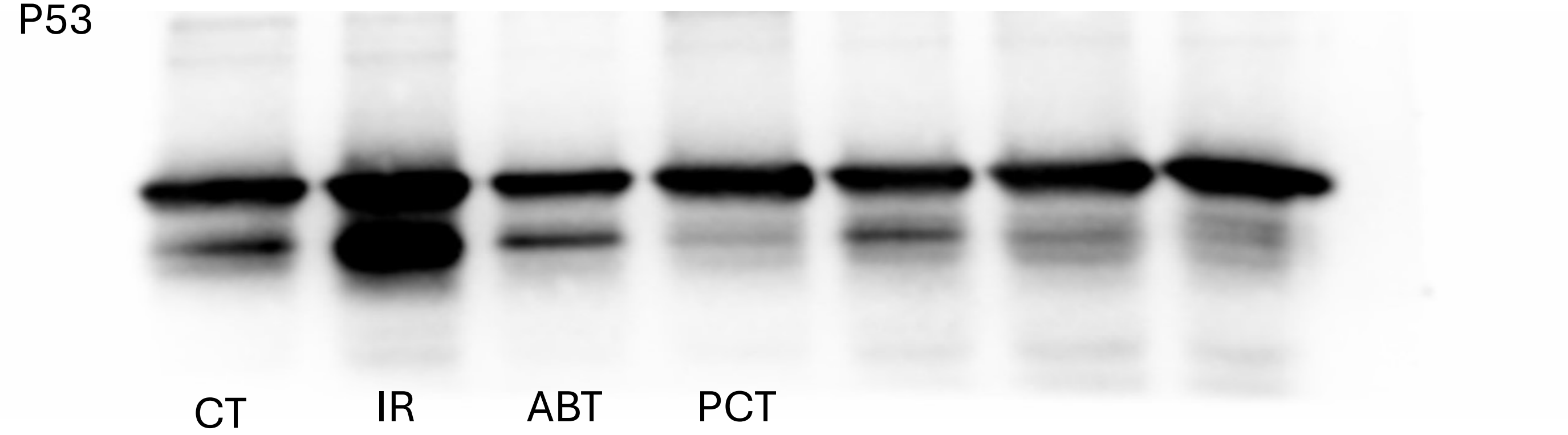

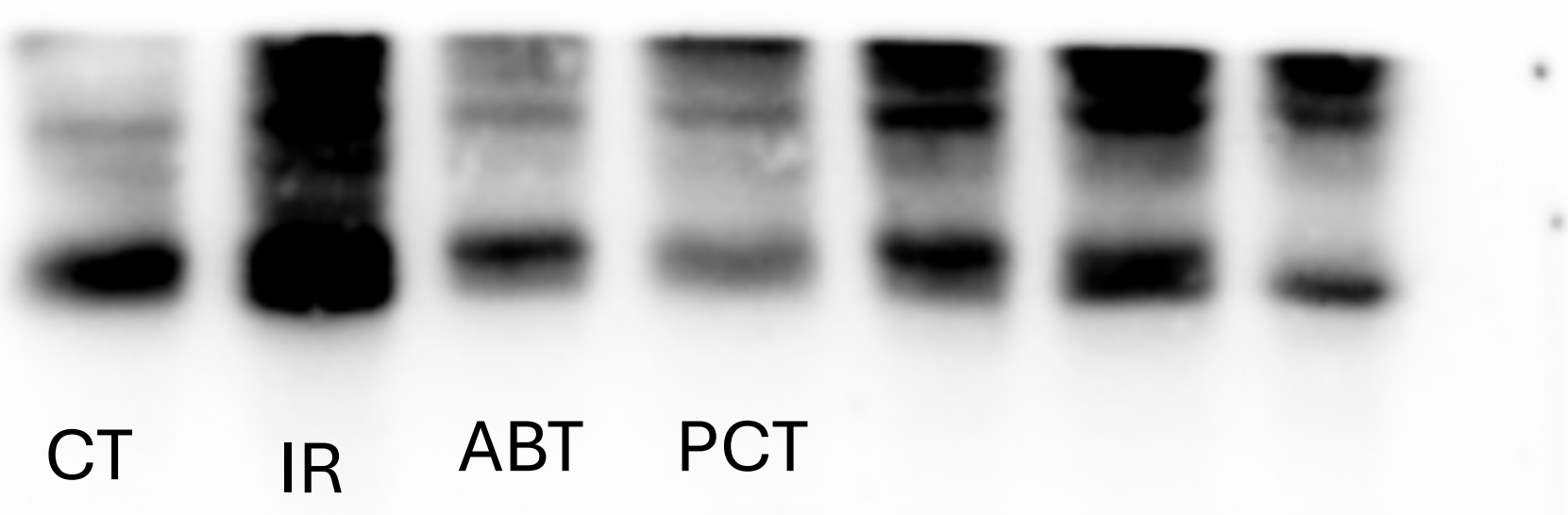

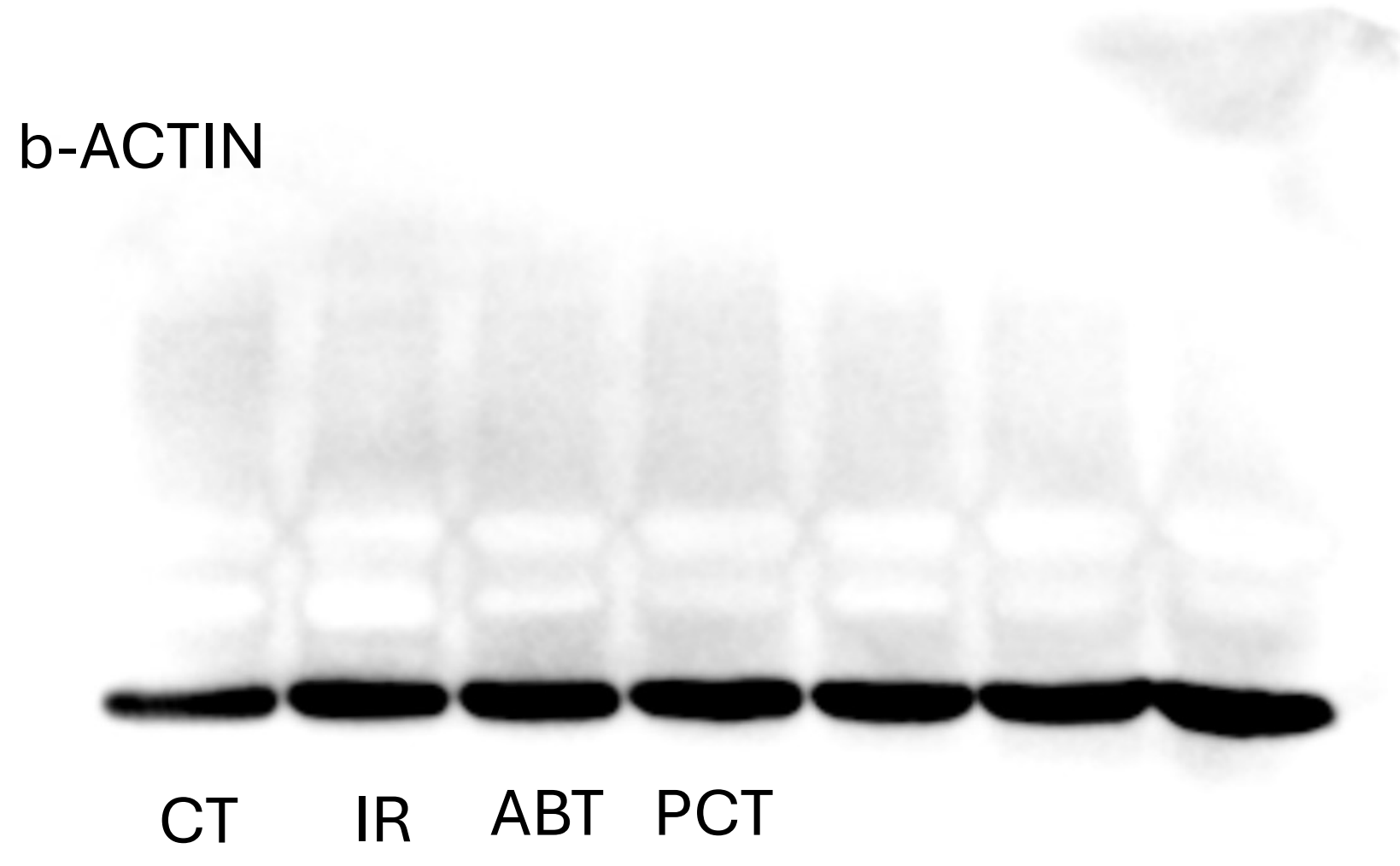
b-ACTIN
CT
IR
ABT
PCT

Figure 7

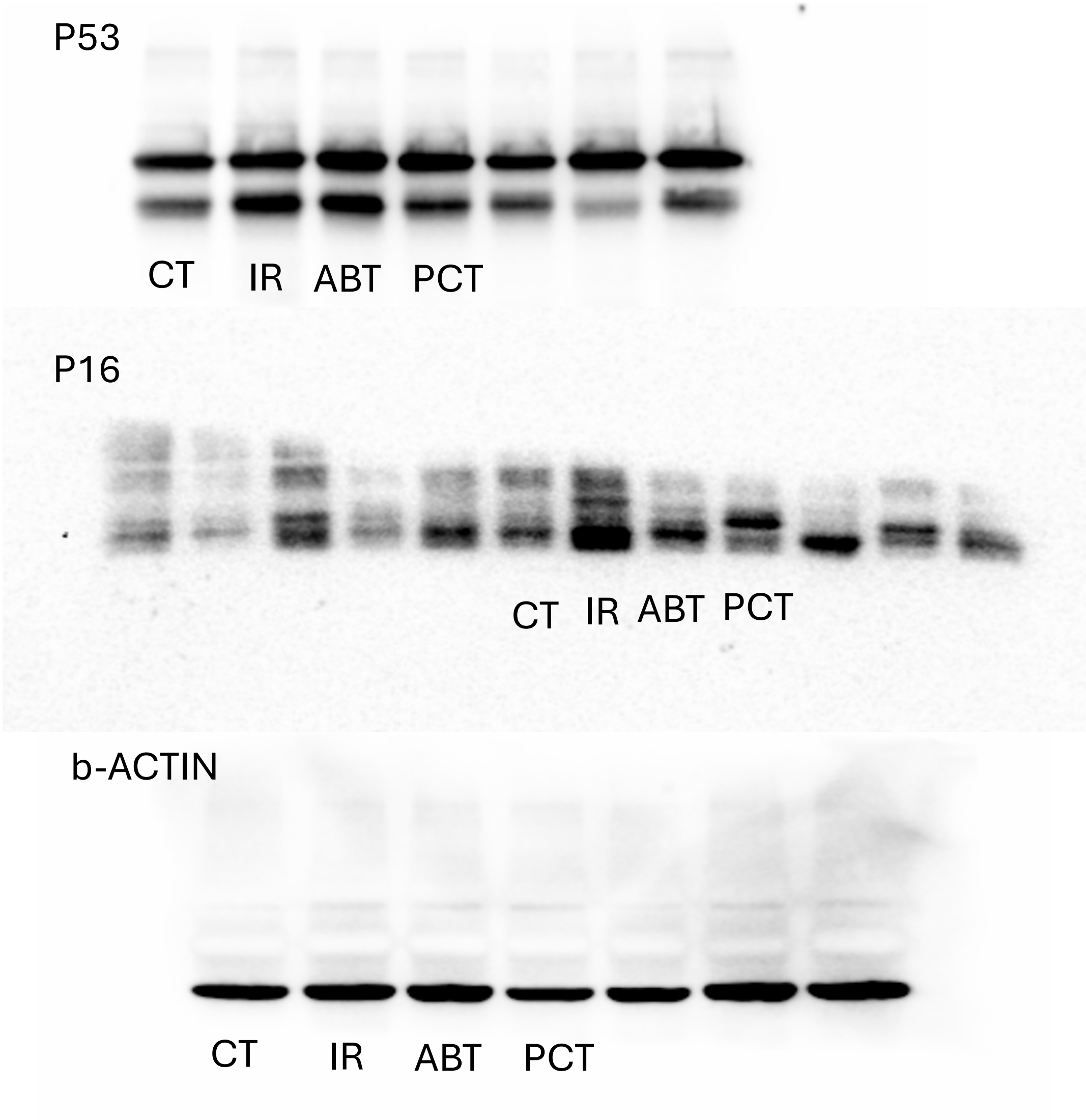

Supplement: Supplementary file 1 [file 41420_2026_3253_MOESM2_ESM.pdf]
