# Supplementary material for: Piceatannol as a safe senotherapeutic: ameliorating senescence-associated markers in a radiation-induced murine model
Source: Cell Death Discov. 2026 Jul 14;12:408. doi: 10.1038/s41420-026-03253-1 (PMC13646293; doi:10.1038/s41420-026-03253-1)
Supplement: Supplementary file 2 [file 41420_2026_3253_MOESM4_ESM.docx]

ELISA and Metabolism kit used in the manuscript

| MARKER | COMPANY | CODE | METHODS | APPLICATION |
| --- | --- | --- | --- | --- |
| Cys-c (Cystatin C) | ElabScience | E-EL-M3024 | Elisa | Kidney |
| Cr (Creatinine) | ElabScience | E-EL-0058 | Elisa | Kidney |
| Urea | ElabScience | E-BC-K329-S | Metabolism Kit | Kidney |
| ALB (Albumin) | ElabScience | E-EL-M30032 | Elisa | liver |
| AST (Aspartate Aminotransferase) | ElabScience | E-EL-M0160 | Elisa | Liver |
| NT-proNBP (N-terminal pro-Brain Natriuretic Peptide) | ElabScience | E-EL- M0834 | Elisa | Heart |
| cTnT/TNNT2 (Troponin T type 2, cardiac) | ElabScience | E-EL-M1801 | Elisa | Heart |
| TNF-α (Tumor Necrosis Factor Alpha) | ElabScience | E-MSEL-M0002 | Elisa | Inflammation |
| IL-6 (Interlukin 6) | ElabScience | E-MSEL-M0001 | Elisa | Inflammation |
| hs-CRP (high-sensitivity C-Reactive Protein) | ElabScience | E-M0677 | Elisa | Inflammation |
| GDF15(Growth Differentiation Factor 15) | ElabScience | E-EL-M0604 | Elisa | Inflammation |

Antibodies used in the manuscript

| ANTIGEN | COMPANY | CODE | APPLICATION | DILUTION | CATEGORY |
| --- | --- | --- | --- | --- | --- |
| RB1 | ElabScience | E-AB-32752 | WB | 1:1000 | Senescence marker |
| P53 | Santa Cruz | SC-126 | WB | 1:3000 | Senescence marker |
| P21 | Santa Cruz | SC-397 | WB | 1:500 | Senescence marker |
| P16 | Santa Cruz | SC-74401 | WB | 1:500 | Senescence marker |
| IGFBP4 | Elabscience | E-AB62793 | WB | 1:500 | SASP marker |
| IGFBP5 | Santa Cruz | SC-6006 | WB | 1:500 | SASP marker |
| IGFBP7 | Cell signaling | 64563 | WB | 1:500 | SASP |
| CD68 | Elabscience | E-AB-66399 | IF | 1:200 | Inflammation |
| β-Actin | Santa Cruz | SC-47778 | WB | 1:20000 | Housekeeping |
| Goat Anti-Rabbit IgG(H+L) (peroxidase/HRP conjugated) | ElabScience | E-AB-1003 | WB | 1:5000 | Secondary antibody |
| Goat Anti-Mouse IgG(H+L) (peroxidase/HRP conjugated) | ElabScience | E-AB-1001 | WB | 1:5000 | Secondary antibody |

Primers used in the manuscript.

| GENE | PRIMER SEQUENCES | TA | SIZE | APPLICATION |
| --- | --- | --- | --- | --- |
| *Ccl2* | 5’- CCAAAGAAGCTGTAGTTT- 3’  3’- AGTTGTAGGTTCTGATCT - 5’ | 56 | 124 | Immune System modulator |
| *Ccl8* | 5-’AGAGAATCAACAATATCCAG - 3’  3’- GATCTCCATGTACTCACT -5’ | 58 | 112 | Immune System modulator |
| *Cxcl1* | 5’ - CGATGGTAGTTCAGTTCTG - 3’  3’- AGTTGGTGACACGATCAT - 5’ | 54 | 108 | Immune System modulator |
| *Cxcl2* | 5’- ACCGAGGAGAGTAGAACA – 3’  3’- TACAGAAGGAGCATTCCG – 5’ | 56 | 129 | Immune System modulator |
| *Il-1* | 5'- ATCTTAATCTGTGCTGTT – 3’  3' - ATTGATTGTGGTTGGTAG – 5’ | 55 | 128 | Immune System modulator |
| *Il-8* | 5’- ACGATGTCTGTGTATTCA -3’  3’- TTCCTTCACTATCGTCAG –5’ | 54 | 111 | Immune System modulator |
| *Tnf* | 5’- CTCTCATCAGTTCTATGG - 3’  3’ – AGTTTCTGTTGGTTGATC - 5’ | 57 | 128 | Immune System modulator |
| *Inha* | 5’ – CTAGGAGGAGGACAGAGTG – 3’  3’ – AGGTATAGGACGAGGTGG – 5’ | 58 | 145 | Immune System modulator |
| *Mmp3* | 5’- AAGATGATGTAGATGGTA – 3’  3’- GAGATGAAACAAGAAACT – 5’ | 57 | 132 | Extra cellular remodelling |
| *Mmp12* | 5’- TAGCAGTCCACCATCAAC – 3’  3’- TCTGACCAAGACCAC- 5’ | 56 | 101 | Extra cellular remodelling |
| *Timp1* | 5’- CTAAGGAACGGAAATTTG - 3’  3’ –AAAGGGACAAATAGATAGG– 5’ | 58 | 141 | Extra cellular remodelling |
| *Igfbp4* | 5’ – ATTCATTCATTCATTCATTC – 3’  3’ – TAACATCTTACACTGCTCCA – 5’ | 55 | 128 | SASP component and/or senescence inducer |
| *Igfbp5* | 5’ – TCAGACAAGACTCCTTCAG – 3’  3’- GTTGTCGTTATTGGTGTAGTG – 5’ | 55 | 110 | SASP component and/or senescence inducer |
| *Igfbp7* | 5’- GAATCCATGAGCCTCTGTAG – 3’  3’- TGGATTGGGTGATTGTGAAATAAT – 5’ | 55 | 105 | SASP component and/or senescence inducer |
| *Cdkn1a* | 5’ – AAGTTGTTAGCCAAGTAA – 3’  3’- CTACATTTACCTTGAAGAC – 5’ | 57 | 146 | Cell cycle |
| *Gapdh* | 5’ -AGCCTCGTCCCGTAGACAAAA -3’  3’ ACGGTGCCATGGAATTTGC 5’ | 56 | 160 | Housekeeping |
